# Editorial: Emerging infections and diseases of herpetofauna

**DOI:** 10.3389/fvets.2022.909616

**Published:** 2022-09-27

**Authors:** Steven J. R. Allain, Amanda L. J. Duffus, Rachel E. Marschang

**Affiliations:** ^1^Durrell Institute of Conservation and Ecology, University of Kent, Canterbury, United Kingdom; ^2^Health of Herpetofauna Communities Research Group, Department of Natural Sciences, Gordon State College, Barnesville, GA, United States; ^3^Laboklin GmbH & Co., KG, Bad Kissingen, Germany

**Keywords:** *Batrachochytrium dendrobatidis*, *B. salamandrivorans*, herpesvirus, ophidiomycosis, ranavirus, reptile, amphibian

We are in the midst of a period of unprecedented global biodiversity declines, which has been dubbed the sixth mass extinction (1–3). While many factors are contributing to these extreme declines, habitat loss and anthropogenic environmental change appear to be the largest drivers for many vertebrates [e.g., mammals, (3)]. There are an increasing number of infectious agents being identified in both amphibians and reptiles around the globe, and more data links infection and disease for many of these emerging pathogens. Human activities also influence infectious disease epidemiology, with interactions between captive animals, those in trade, and wild animals affecting infection dynamics. One important factor in increasing our understanding of these interactions is a better overview of the infection status of both wild and captive amphibian and reptile populations and communities.

The emergence of fungal infections and their resultant mycoses, has, for over the past two decades, been associated with enigmatic declines in several taxa. In amphibians, the emergence of chytridiomycosis, the disease caused by infection with *Batrachochytrium dendrobatidis* (*Bd*), was first described in 1998 (4) and has now contributed to the declines of over 500 species and the extinction of at least 90 [(5), but see Lambert et al. (6)]. A second amphibian chytrid, *Batrachochytrium salamandrivorans* (*Bsal*), was described by Martel et al. (7) when it caused unprecedented declines in European Fire Salamanders (*Salamandra salamandra*). It now threatens salamander populations around the globe, although it is currently limited to Europe and Asia.

A large portion of this Research Topic on emerging infections and diseases of herpetofauna is appropriately dedicated to studies and reviews of data on *Bd* and *Bsal*. Olson et al. detail the importance of global tracking databases for *Bd* and the transition of Bd-maps.net to AmphibianDisease.org, which tracks both *Bd* and *Bsal* infections (also see Koo et al.). Olson et al. also examine the occurrence of *Bd* in amphibians around the globe. Koo et al. discuss the utility, functionality, and importance of the AmphibianDisease.org database.

Urbina et al. examine the short- and long-term effects of *Bd* exposure on embryos. Sheets et al. examine the phenotypic responses of several strains of *Bd* to a temperature gradient. Alvarado-Rybak et al. investigate a *Bd*-associated mortality event in native Chilean frogs in a captive breeding program. Cowgill et al. study the effects of *Bd* at the community level in the Pacific Northwest of the USA. Belasen et al. perform a meta-analysis that supports previous findings that historical coexistence between host and endemic *Bd* lineages is associated with less disease and mortality, but that more recent coexistence with the *Bd* global pandemic lineage is not. These studies highlight the diversity of Research Topics that continue to be investigated.

Fungal pathogens are also a cause for concern in reptile medicine and conservation. In snakes, *Ophidiomyces ophidiicola* (*Oo*), the causative agent of ophidiomycosis (formerly known as snake fungal disease) has caused disease outbreaks in wild and captive animals (8). In North America, *Oo* has been associated with the decline of several snake species (9), and it has also been detected in wild European snakes (10). Understanding the distribution and impact of *Oo* globally is an important Research Topic that clearly requires additional study. In this issue, Davy et al. explore *Oo* infections in Ontario, Canada, and their results suggest that earlier assertions that *Oo* is endemic and widespread in the area are likely correct.

Viruses also pose a large threat to amphibians and reptiles around the globe. Herpesviruses have been reported in both amphibians and reptiles [e.g., Okoh et al.; (11)]. In amphibians, proliferative dermatitis has been associated with newly described herpesviruses in common frogs (*Rana temporaria*) and common toads (*Bufo bufo*) (11, 12), but relatively little is known about the biology of herpesviruses in amphibians. In reptiles, herpesviruses have been described in a wide range of species, most commonly in chelonians, and their clinical impact appears to depend on many different factors. In recent years, the number of genetically distinct herpesviruses described in reptiles has grown rapidly and includes descriptions in wild and captive animals (Okoh et al.). The overview in this issue of herpesviruses detected in captive chelonians in Europe in recent years (Leineweber et al.) indicates a complex dynamic, with the pet trade playing a role in the dissemination of viruses to new parts of the world.

Ranaviruses are globally distributed pathogens of amphibians and reptiles [see Duffus et al. (13)]. Causing infection and disease in many species, ranaviruses are a threat to populations of amphibians and reptiles around the globe. We are still determining the real geographic range and number of species affected (e.g., Box et al.). Ranaviruses are a group of pathogens that can cause population declines [e.g., *R. temporaria*; ((14) Bosch et al.)] and in models can persist within populations while the declines happen [e.g., *R. temporaria*; (15)] or completely decimate the tadpole population [e.g., *Lithobates sylvaticus*; (16)]. Additionally, we have little knowledge of how ranaviruses act in amphibian communities. Bienentreu et al. investigate how ranaviruses act in low-diversity amphibian communities in northern Canada. Importantly, they find that species richness impacts infection prevalence, suggesting that an increased number of species in a community has an amplification effect on infection rates.

Viruses in the order *Nidovirales*, family *Tobaniviridae*, and subfamily *Serpentovirinae*, were first reported as a cause of severe respiratory disease in pythons in 2014 [(17); see Marschang et al. (18) for an overview]. These viruses have since been shown to be a common cause of disease in many snake species with high prevalences reported in captive pythons in the United States and Europe. Similar viruses have been described in association with respiratory disease in other squamates including wild-caught shingleback lizards (*Tiliqua rugosa*) in Australia (19), and captive veiled chameleons (*Chamaeleo calyptratus*) in the United States (20). A related virus was also found during a large die-off of Bellinger River snapping turtles (*Myuchelys georgesi*) in Australia (21). Although the virus isolated from those animals was not proven to have caused the mortalities, it was hypothesized to have played an important role. The episode appeared to impact the viability of the wild population. The fast pace at which nidoviruses have been discovered in reptile hosts and the changes and advances made in recent years to the taxonomy of this group make the review of this topic published by Parrish et al. in this issue particularly timely.

In addition to the growing knowledge base on the diversity and impacts of these fungi and viruses, new potential pathogens are being described in growing numbers in herpetofauna [see e.g., (22)]. In this issue, Tillis et al. describe a novel species of *Actinomyces* associated with granulomatous disease in captive ball pythons (*Python regius*). Their study indicates a possible sexual transmission and impact on the breeding of this popular species. New infectious disease threats in herpetofauna are likely to continue to be discovered in the future. In addition to these discoveries, researchers continue to investigate the complex connections between specific pathogens, diverse hosts, life stages, environmental factors, disease development, and pathogen dissemination. The diversity of topics covered within this special issue is representative of the infectious disease threats that amphibian and reptile populations face around the globe both in the wild and in captivity. This provides an important synopsis of current knowledge, while also helping to expand that information base for future researchers.

## Author contributions

All authors listed have made a substantial, direct, and intellectual contribution to the work and approved it for publication.

## Funding

SA was supported by the Natural Environment Research Council through the EnvEast Doctoral Training Partnership (Grant No. NE/L002582/1).

## Conflict of interest

Author RM is employed by Laboklin GmbH & Co. KG. The remaining authors declare that the research was conducted in the absence of any commercial or financial relationships that could be construed as a potential conflict of interest.

## Publisher's note

All claims expressed in this article are solely those of the authors and do not necessarily represent those of their affiliated organizations, or those of the publisher, the editors and the reviewers. Any product that may be evaluated in this article, or claim that may be made by its manufacturer, is not guaranteed or endorsed by the publisher.
